# Immunity Traits in Pigs: Substantial Genetic Variation and Limited Covariation

**DOI:** 10.1371/journal.pone.0022717

**Published:** 2011-07-29

**Authors:** Laurence Flori, Yu Gao, Denis Laloë, Gaëtan Lemonnier, Jean-Jacques Leplat, Angélique Teillaud, Anne-Marie Cossalter, Joëlle Laffitte, Philippe Pinton, Christiane de Vaureix, Marcel Bouffaud, Marie-José Mercat, François Lefèvre, Isabelle P. Oswald, Jean-Pierre Bidanel, Claire Rogel-Gaillard

**Affiliations:** 1 INRA, UMR 1313 de Génétique Animale et Biologie Intégrative, Domaine de Vilvert, Jouy-en-Josas, France; 2 CEA, DSV, IRCM, SREIT, Laboratoire de Radiobiologie et Etude du Génome, Domaine de Vilvert, Jouy-en-Josas, France; 3 AgroParisTech, Laboratoire de Génétique Animale et Biologie Intégrative, Domaine de Vilvert, Jouy-en-Josas, France; 4 INRA, UR66 de Pharmacologie-Toxicologie, 180 chemin de Tournefeuille, BP 93173, Toulouse, France; 5 INRA, UR892 de Virologie et Immunologie Moléculaires, Jouy-en-Josas, France; 6 INRA, Station de contrôle de performances, UE450, Le Rheu, France; 7 IFIP, Pôle amélioration de l'animal, La Motte au Vicomte, France; South Texas Veterans Health Care System, United States of America

## Abstract

**Background:**

Increasing robustness via improvement of resistance to pathogens is a major selection objective in livestock breeding. As resistance traits are difficult or impossible to measure directly, potential indirect criteria are measures of immune traits (ITs). Our underlying hypothesis is that levels of ITs with no focus on specific pathogens define an individual's immunocompetence and thus predict response to pathogens in general. Since variation in ITs depends on genetic, environmental and probably epigenetic factors, our aim was to estimate the relative importance of genetics. In this report, we present a large genetic survey of innate and adaptive ITs in pig families bred in the same environment.

**Methodology/Principal Findings:**

Fifty four ITs were studied on 443 Large White pigs vaccinated against *Mycoplasma hyopneumoniae* and analyzed by combining a principal component analysis (PCA) and genetic parameter estimation. ITs include specific and non specific antibodies, seric inflammatory proteins, cell subsets by hemogram and flow cytometry, *ex vivo* production of cytokines (IFNα, TNFα, IL6, IL8, IL12, IFNγ, IL2, IL4, IL10), phagocytosis and lymphocyte proliferation. While six ITs had heritabilities that were weak or not significantly different from zero, 18 and 30 ITs had moderate (0.1<h2≤0.4) or high (h2>0.4) heritability values, respectively. Phenotypic and genetic correlations between ITs were weak except for a few traits that mostly include cell subsets. PCA revealed no cluster of innate or adaptive ITs.

**Conclusions/Significance:**

Our results demonstrate that variation in many innate and adaptive ITs is genetically controlled in swine, as already reported for a smaller number of traits by other laboratories. A limited redundancy of the traits was also observed confirming the high degree of complementarity between innate and adaptive ITs. Our data provide a genetic framework for choosing ITs to be included as selection criteria in multitrait selection programmes that aim to improve both production and health traits.

## Introduction

Increasing robustness by improving resistance/tolerance to pathogens is an important selection objective in most livestock species, particularly in pigs. In the past 30 years, selection for growth, carcass leanness, meat quality and prolificacy, combined with stringent sanitary rules, vaccination and use of antibiotics, has been highly effective in pigs [1]. Since the early 2000's, prophylactic use of antibiotics as growth promoters has been forbidden by European legislation. As a result, the health status of numerous farms has deteriorated, leading to an increase in the therapeutic use of antibiotics. Indeed, animals highly selected for production traits may be more susceptible to pathogens or less able to maintain performance after infection. Deterioration of the global health status may also be due to environmental trends. In this context, including health traits in existing breeding schemes using direct and/or indirect strategies is an emerging trend in pig breeding. Direct strategies target animal resistance/tolerance to specific pathogens but may result in increased susceptibility to other diseases [2], [3]. Alternatively, an indirect and putatively more global approach focuses on immune traits (ITs) providing a measure of immune capacity (i.e. immunocompetence) and hopefully predicting the responses to pathogens in general [4].

The choice of relevant ITs is further based on knowledge of the immune system. This highly interactive and cooperative system is classically separated into two arms referred to as innate and adaptive, which produce a combined response. Innate immunity is the first line of defence. Its activation is non pathogen-specific and depends on the recognition of evolutionarily conserved pathogen-associated molecular patterns such as lipopolysaccharides constituting bacterial cell walls [5]. Innate immunity involves physical barriers, innate immune cells such as dendritic cells (DCs), monocytes, natural killers (NK cells) or γδ T lymphocytes, and inflammatory cytokines such as IL1B, IL6 and TNF. Adaptive immunity is antigen-specific and requires the recognition of specific “non-self” antigens via a process of antigen presentation and results in an immunological memory. Adaptive immunity is divided into cell- and humoral-mediated immunity with different effector functions [6].

In order to include ITs in a breeding plan to improve pig immunocompetence, the genetic and phenotypic parameters of the different ITs need first to be estimated. Several studies in swine, mice, poultry and cattle demonstrated the possibility of selecting animals with high or low immune response (IR) as characterized by one or a few ITs [2], [7], [8], [9], [10]. A study on Yorkshire pigs selected for eight generations for high and low adaptive IR (HIR and LIR, respectively) on an index combining four standardized measures of specific antibodies and cell-mediated IR, after stimulation with specific antigens (bacillus Calmette-Guérin and hen egg white lysozyme), has revealed that HIR and LIR animals differ in response to immunization and infection [2], [11], [12], [13], [14]. Other studies have also shown that various innate and adaptive ITs are genetically controlled. For example, variation in innate ITs, such as NK cells, monocytes, interferon α (IFNα) production or phagocytosis [15], [16], [17] is heritable and several adaptive ITs have moderate to high heritability values including total white blood cells (WBC), CD4^+^ T lymphocyte, CD8α^+^ T lymphocyte and B lymphocyte subsets [15], [16], [17], delayed-type hypersensitivity reaction [15], [18], lymphocyte proliferative response [15], interleukin-2 (IL2) production by lymphocytes [15] and antibody response [12], [15], [18], [19]. Clapperton and colleagues have also reported that variation in acute phase protein levels is heritable [16], [17]. Finally, several significant QTLs for total leukocyte count ([20], [21]; AnimalQTLdb, http://www.animalgenome.org/cgi-bin/QTLdb/index), mitogen-induced proliferation [20], antibody response [20], [22], cytokine production (IL10 and IFNγ) [23], complement activity [22], and acute phase protein serum concentration [22] have been detected and mapped to different pig chromosomes. Taken together these data demonstrate that variation in some ITs is under genetic control. However, most of the results reported so far have targeted a limited number of traits and very few studies have combined innate and adaptive ITs.

Our global goal is to identify immunocompetence traits for inclusion in selection schemes aiming to improve both zootechnical performances and health traits in pigs. For this purpose, we have launched a genetic and genomic study of numerous ITs covering innate and adaptive IR [24]. In this report, we present the results of a global genetic study, combining principal component analysis (PCA), and genetic parameter estimation applied to a large number of innate and adaptive ITs in a pig population vaccinated against *Mycoplasma hyopneumoniae (M. hyopneumoniae).*

## Results

### ITs and descriptive statistics

**Figure 1:**
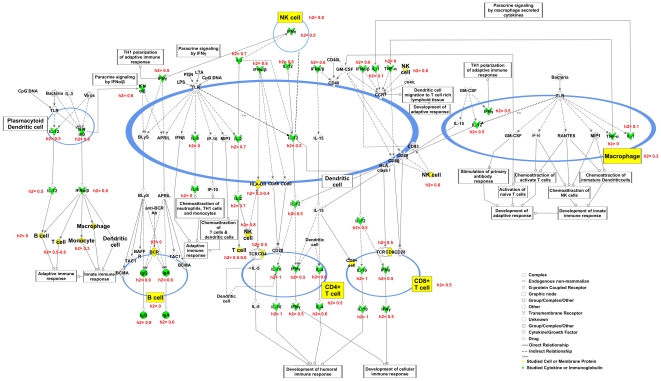
Position of the measured ITs on a global scheme of immunity. The global scheme of immunity was obtained using *Ingenuity Pathway Analysis* software v 8.8 (Ingenuity Systems Inc., USA, http://www.ingenuity.com/). Cellular subsets and membrane proteins considered in this study are in yellow and cell activity traits in green. For each trait, the heritability estimate rounded to one decimal place is shown in red.

A set of 54 ITs was measured on a population of 443 pigs three weeks after vaccination against *M. hyopneumoniae* (Tables 1 and 2; Table S1; Figure 1). These ITs comprise either traits related to IR (phagocytosis, lymphocyte proliferation, cytokine production after *in vitro* stimulations, levels of total and specific antibodies, levels of acute phase proteins) or traits related to total leukocyte and leukocyte subpopulation counts. The various characteristics and descriptive statistics of the traits measured on each animal of the studied population (n = 383 to 442) are summarized in Tables 1 and 2. Among the cell-mediated ITs evaluated after diverse stimulations, higher responses in cytokine levels were observed after phorbol myristate acetate (PMA)-Ionomycin (PMAIONO) stimulation compared to lipopolysaccharide (LPS) and concanavalin A (CONA) stimulations, except for IL2 production. Conversely, a higher lymphocyte proliferation was detected after CONA and PMAIONO stimulations than after LPS stimulation.

**Table 1:** Descriptive statistics and heritability estimates of the ITs contributing to global and adaptive immunity.

Type of traits	Trait symbol	N&	Mean	sd	CV	h^2^ (se)	95%CI of h^2^	h^2^ levels#
**Global immunity**	**WBC** *	**440**	**18.0**	**5.2**	**0.28**	**0.73 (0.20)**	**[0.33–1.12]**	**High**
	**LYM** *	**440**	**12.7**	**3.7**	**0.29**	**0.72 (0.21)**	**[0.30–1.13]**	**High**
**Cell-mediated adaptive immunity**	**IL4-CONA**	**434**	**117.9**	**120.5**	**1.02**	**0.55 (0.20)**	**[0.10 – 0.94]**	**High**
	**IL4-PMAIONO** *	**438**	**1360.2**	**1804.7**	**1.32**	**0.61 (0.22)**	**[0.10– 1.00]**	**High**
	IL4-LPS	438	77.6	80.9	1.04	0.15 (0.18)	[0 .00– 0.50]	Moderate
	IL10-CONA	438	305.0	404.4	1.32	0.50 (0.21)	[0.00 – 0.91]	High
	**IL10-PMAIONO** *	**438**	**1444.7**	**1492.7**	**1.03**	**1.00 (0.21)**	**[0.50 – 1.00]**	**High**
	IL10-LPS	438	506.9	610.4	1.20	0.35 (0.19)	[0.00 – 0.72]	Moderate
	IFNG-CONA	437	516.9	552.2	1.06	0.41 (0.19)	[0.00 – 0.78]	Moderate
	**IFNG-PMAIONO** *	**437**	**56799**	**79326**	**1.39**	**0.52 (0.20)**	**[0.10 – 0.91]**	**High**
	IFNG-LPS	438	530.4	692.6	1.30	0.00 (0.17)	[0.00 – 0.33]	Weak
	IL2-CONA	436	9827.3	8707.5	0.88	0.88 (0.55)	[0.00 – 1.00]	High
	**IL2-PMAIONO** *	438	**7159.5**	**7257.6**	**1.01**	**0.71 (0.21)**	**[0.2 – 1.00]**	**High**
	**IL2-LPS**	438	**6696.1**	**6152.6**	**0.91**	**0.91 (0.19)**	**[0.50 – 1.00]**	**High**
	CD4^-^ CD8^+^ *	383	13.8	7.1	0.51	0.50 (0.23)	[0.00 – 0.95]	High
	**CD4^+^ CD8^+^** *	**383**	**3.1**	**2.0**	**0.63**	**0.64 (0.22)**	**[0.20 – 1.00]**	**High**
	**CD4^+^ CD8^-^** *	**431**	**5.1**	**3.0**	**0.57**	**0.54 (0.21)**	**[0.10 – 0.95]**	**High**
	PROLIF-CONA	392	28.1	33.9	1.20	0.36 (0.20)	[0.00– 0.75]	Moderate
	PROLIF-PMA *	392	30.5	48.3	1.57	0.27 (0.20)	[0.00 – 0.66]	Moderate
	PROLIF-LPS	392	0.3	0.5	1.29	0.31(0.19)	[0.00 – 0.68]	Moderate
**Humoral-mediated adaptive immunity**	IgM^+^ *	384	22.1	12.3	0.55	0.00 (0.21)	[0.00 – 0.41]	Weak
	IgG-Mh *	441	0.3	1.2	3.90	0.12 (0.19)	[0.00 – 0.49]	Moderate
	**IgA** *	**441**	**44.7**	**25.2**	**0.56**	**0.68 (0.20)**	**[0.20 – 1.00]**	**High**
	**IgG** *	**436**	**68.9**	**37.1**	**0.53**	**0.92 (0.21)**	**[0.50 – 1.00]**	**High**
	IgM*	441	74.3	55.6	0.74	0.31 (0.18)	[0.00–0.66]	Moderate

& N  =  number of progeny measured for each IT.

*ITs taken into account in PCA and for which genetic correlations have been estimated.

# high (h^2^>0.4), moderate (0.1<h^2^<0.4), weak (h^2^≤0.1).

ITs with significant h^2^ (95CI excluding 0) emboldened.

**Table 2:** Descriptive statistics and heritability estimates of the ITs contributing to innate immunity and of other hematological traits.

Type of traits	Trait symbol	N&	Mean	sd	CV	h^2^ (se)	95%CI of h2	h^2^ levels#
**Innate immunity**	MON *	440	1.0	0.4	0.36	0.38 (0.20)	[0.00 – 0.77]	Moderate
	**NEU** *	**440**	**3.9**	**1.8**	**0.45**	**0.61 (0.20)**	**[0.20 – 1.00]**	**High**
	**EOS** *	**440**	**0.2**	**0.1**	**0.40**	**0.80 (0.21)**	**[0.30 – 1.00]**	**High**
	IL1B *	442	6150.7	5581.8	0.90	0.12 (0.19)	[0.00– 0.49]	Moderate
	IL8 *	442	10663	12666	1.18	0.00 (0.17)	[0.00 – 0.33]	Weak
	TNF *	442	2091.6	1619.9	0.77	0.00 (0.19)	[0.00 – 0.37]	Weak
	IL6 *	439	222.2	435.5	1.95	0.11 (0.19)	[0.00 – 0.48]	Moderate
	**IFNA** *	**404**	**394.1**	**628.2**	**1.59**	**0.60 (0.23)**	**[0.10 – 1.00]**	**High**
	**IL12** *	**442**	**740.1**	**785.4**	**1.06**	**0.51 (0.20)**	**[0.10 – 0.90]**	**High**
	CD16^-^ CD2^+^	432	18.4	8.6	0.46	0.32 (0.21)	[0.00 – 0.73]	Moderate
	**CD16^+^ CD2^+^** *	**432**	**8.5**	**5.6**	**0.65**	**0.81 (0.20)**	**[0.40 – 1.00]**	**High**
	CD16^+^ CD2^-^	432	16.6	11.2	0.67	0.05 (0.16)	[0.00 – 0.36]	Weak
	MHCII^-^ CD172A^+^	384	12.6	9.1	0.71	0.25 (0.21)	[0.00 – 0.66]	Moderate
	MHCII^+^ CD172A^+^ *	384	5.7	3.1	0.54	0.48 (0.21)	[0.00 – 0.89]	High
	MHCII^+^ CD172A^-^	432	19.1	9.5	0.49	0.39 (0.20)	[0.00 – 0.78]	Moderate
	CD16^-^ CD172A^+^	384	1.5	1.2	0.75	0.30 (0.22)	[0.00 – 0.73]	Moderate
	CD16^+^ CD172A^+^ *	384	17.4	10.7	0.61	0.21 (0.20)	[0.00 – 0.60]	Moderate
	**CD16^+^ CD172A^-^**	**432**	**9.0**	**7.2**	**0.79**	**0.74 (0.20)**	**[0.30 – 1.00]**	**High**
	CD16^-^ MHCII^+^	432	15.4	7.2	0.46	0.02 (0.17)	[0.00 – 0.35]	Weak
	**CD16^+^ MHCII^+^** *	**431**	**9.6**	**5.8**	**0.60**	**0.71 (0.19)**	**[0.30 – 1.00]**	**High**
	CD16^+^ MHCII^-^	432	14.8	9.7	0.65	0.17 (0.20)	[0.00 – 0.56]	Moderate
	**TCRγδ^+^** *	**431**	**12.4**	**7.9**	**0.63**	**0.69 (0.21)**	**[0.20– 1.00]**	**High**
	**PHAG** *	**414**	**33.7**	**10.2**	**0.30**	**0.62 (0.22)**	**[0.10 – 1.00]**	**High**
	**HAPT** *	**440**	**0.7**	**1.6**	**2.05**	**0.55 (0.21)**	**[0.10 – 0.96]**	**High**
	CRPT *	440	106.5	167.1	1.56	0.18 (0.18)	[0.00 – 0.53]	Moderate
**Other hematological traits**	RBC	440	6.2	0.5	0.07	0.43 (0.20)	[0.00 – 0.82]	High
	**HT**	**440**	**30.8**	**4.8**	**0.15**	**0.57 (0.03)**	**[0.50 – 0.62]**	**High**
	RDW	440	14.8	1.5	0.10	0.70 (nd)	nd	High
	**PLT**	**440**	**539.3**	**257.2**	**0.47**	**0.56 (0.19)**	**[0.10 – 0.93]**	**High**

& N  =  number of progeny measured for each IT.

*ITs taken into account in PCA and for which genetic correlations have been estimated.

# high (h^2^>0.4), moderate (0.1<h^2^<0.4), weak (h^2^≤0.1).

ITs with significant h^2^ (95CI excluding 0) emboldened.

Ample phenotypic variation was observed for most traits. The coefficient of variation (CV) was equal to 0.8 on average and ranged from 0.07 to 3.9 (Tables 1 and 2). Limited dispersion (CV≤0.9) was observed for traits derived from hemograms, cell subsets characterized by fluorescence-activated cell sorting (FACS), phagocytosis capacity and non-specific immunoglobulins. Moderate dispersion (0.9<CV≤1.5) was observed for most cytokines produced *in vitro* except for tumour necrosis factor α (TNFα) and mitogen proliferation-related traits. Finally, the CV for seric C reactive protein (CRP) and haptoglobin (HAPT) levels were close to 1.6 and 2, respectively. The highest CV (3.9) was obtained for the specific IgGs directed against *M. hyopneumoniae*. These data clearly indicate that the seric inflammatory protein levels and the specific IgGs had the greatest phenotypic variance in our study.

### Multivariate dispersion and phenotypic correlations

**Figure 2:**
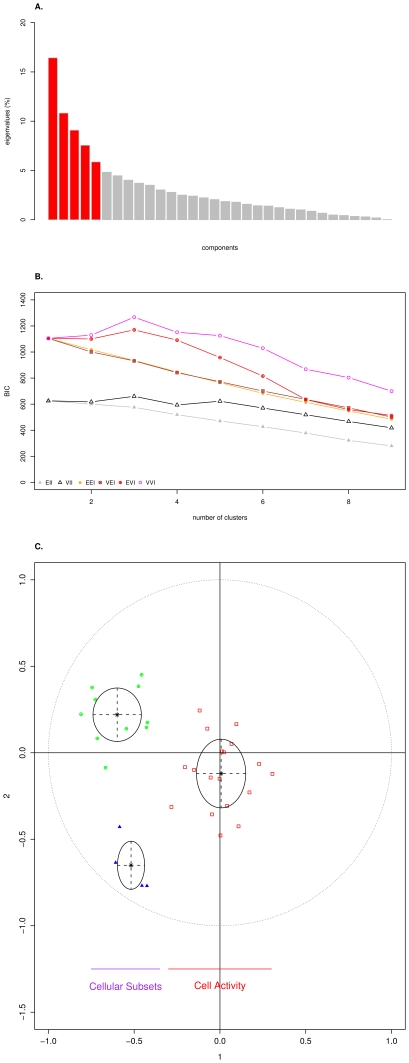
Normed PCA on 32 ITs. A. Histogram of eigenvalues. The five first components, which explain more than 50% of the total variance, are in red. B. Plot of the Bayesian Information Criterion (BIC) calculated with different models according to number of clusters. Six models are compared: EII (spherical with equal volume and equal shape), VII (spherical with variable volume and equal shape), EEI (diagonal with equal shape and equal volume), VEI (diagonal with variable shape and equal volume), EVI (diagonal with equal shape and variable volume), VVI (diagonal with variable shape and variable volume). C. First factorial plan (1: first component, 2: second component) with three clusters identified by multivariate normal mixture modelling and model-based clustering taking into account the 32 components (clusters K1, K2 and K3 are in blue, green and red, respectively).

In order to analyse the factors causing the variation, we performed a normed PCA with 32 traits (Tables 1 and 2). For cell-mediated adaptive IR, we included only those traits related to cytokine production and lymphocyte proliferation after PMA-ionomycin stimulation. For innate ITs, we included 10 FACS-characterized cell subtypes including the percentages of B lymphocytes (IgM^+^), γδ T lymphocytes (TCRγδ^+^), three subsets of αβ T lymphocytes (CD4^+^ CD8^+^, CD4^-^ CD8^+^ and CD4^+^ CD8^-^), NK cells (CD16^+^ CD2^+^) and three monocyte subsets (CD16^+^ CD172A^+^, CD16^+^ MHCII^+^, MHCII^+^ CD172A^+^). We excluded from the analysis four haematological traits not directly involved in immunity: red blood cell count (RBC), hematocrit (HT), red blood cell distribution width (RDW) and platelet count (PLT). The percentage of variance (inertia) explained by the first five components was over 50% (Figure 2A). Each of these five components explained more than 5% of the total variance and the first two components accounted for 16.4 and 10.8% of the total variance, respectively. Taking into account the 32 components from PCA, multivariate normal mixture modelling and model-based clustering (see Materials and Methods) were used to identify clusters of ITs. The highest Bayesian Information Criterion (BIC) was obtained using the diagonal model with variable shape and variable variance (VVI in pink on Figure 2B) and K = 3 (first factorial plan on Figure 2C). No parameter was located near the correlation circle indicating that the phenotypic correlations between ITs are globally weak. A first cluster (K1 in blue on Figure 2C) groups together four hemogram–derived cell counts: white blood cell count (WBC), lymphocyte count (LYM), monocyte count (MON) and neutrophil count (NEU). This cluster is representative of total cell number traits despite the eosinophil count (EOS) not being included. A second cluster (K2 in green on Figure 2C) groups together all the traits related to FACS-characterized leukocyte subpopulations (expressed as the percentage of cells with one or two surface antigens) except γδ T lymphocytes (TCRγδ^+^), with a cell response parameter (IL10-PMAIONO) and the seric level of haptoglobin (HAPT). This cluster can be considered as representative of the leukocyte subsets. A third cluster (K3 in red on Figure 2C) includes all other cell response traits, one hemogram-derived cell count (EOS) and one FACS-characterized leukocyte subpopulation. Note that K1 and K2 related traits, which mainly correspond to cell subsets and explain around 25% of the phenotypic variance, show clear clustering (Figure 2C). These traits are grouped on the first PCA axis and separated on the second axis. Traits belonging to K3, representative of cell activity (cytokine production, phagocytosis and antibody production), are more spread out on the other axes (data not shown). Interestingly, cluster analysis did not highlight any cluster of innate or adaptive ITs.

The estimation of phenotypic correlations (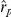) with WOMBAT confirmed that the ITs are weakly correlated, except i) among a few cell count traits (WBC, LYM, MON, NEU), and ii) between cell count traits and a few leukocyte subsets (WBC, LYM, CD16^+^ CD2^+^ and CD4^-^ CD8^+^) for which 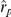 greater than 0.4 were estimated (Table S2; Figure S1). Weak 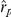 were mainly positive (330) with 166 negative. No strongly negative 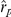 (≤−0.4) were found. Taken together, PCA and estimations of phenotypic correlations showed that the level of redundancy between the different immune parameters was limited.

### A broad range of ITs is heritable

Heritability estimates of the 54 analyzed ITs was equal to 0.45 on average (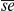  = 0.20; Table 1 and 2). Thirty, 18 and six traits were highly (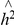
> 0.4), moderately (0.1<
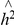
≤0.4) and weakly/not (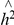
≤ 0.1) heritable, respectively. Moreover, among the highly heritable traits, 24 had a 95% confidence interval (95CI) of heritability that did not overlap zero. The average estimated heritability values of ITs measured directly on blood (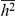 =  0.42, 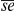  = 0.19) or by *in vitro* tests (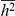 =  0.50, 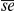  = 0.22) did not significantly differ. Overall, average estimated heritabilities were equal to 0.38 (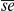 = 0.19) for innate ITs, 0.40 (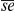  = 0.19) for humoral adaptive ITs and 0.51 (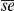  = 0.22) for cellular adaptive ITs. For the large set of adaptive ITs, the mean heritability was 0.48 (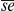  = 0.21). No significant difference in average heritability values was detected either between group of ITs qualifying the innate and adaptive immunity or the humoral and cellular adaptive immunity. In addition, an equivalent proportion of innate and adaptive ITs had significant heritability values. Indeed, 40% (10/25), 40% (2/5) and 44% (8/18) of the innate, humoral-mediated adaptive and cellular-mediated adaptive ITs, respectively, had a 95CI of h^2^ that did not overlap zero. Focussing on the three clusters identified with PCA, the average heritability values of traits included in the clusters K1, K2 and K3 reached 0.61 (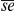  = 0.20), 0.54 (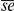  = 0.20) and 0.43 (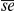  = 0.20), respectively.

Among the traits involved in cell-mediated immunity, variation in αβ T lymphocyte (CD4^-^ CD8^+^, CD4^+^ CD8^+^ and CD4^+^ CD8^-^ cells) counts was highly heritable. Heritability estimates of the three cytokine (IL4, IL10, IFNG) levels were moderate to high after PMAIONO and CONA stimulations and weak to moderate after LPS stimulation. For those cytokines induced by CONA or LPS stimulation, confidence interval (95CI) for the heritabilities overlapped zero, except for IL4-CONA. IL2 production after PMAIONO, CONA and LPS stimulations gave high estimates of heritability significantly different from zero for IL2-PMAIONO and IL2-LPS. Proliferation measurements after various stimulations (PROLIF-CONA, PROLIF-PMAIONO, PROLIF-LPS) provided moderate estimates of heritability not significantly different from zero. Among the traits involved in humoral-mediated adaptive immunity, heritabilities for total IgG and IgA antibody levels were higher than for total IgM and specific antibodies, and weak 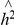 values were obtained for B lymphocyte count (IgM^+^ cells).

Heritability for innate ITs such as i) total cell number (EOS and NEU), ii) leukocyte subsets (CD16^+^ CD2^+^ cells, CD16^+^ CD172A^-^ cells, CD16^+^ MHCII^+^ cells and TCRγδ^+^ lymphocytes), iii) cytokine production (IFNA and IL12), and iv) phagocytosis were high and significantly different from zero. In addition, several innate ITs showed weak to moderate heritability, including pro-inflammatory cytokines (IL1B, IL8, TNF and IL6), MON, CD16^-^ CD2^+^ cells, CD16^+^ CD2^-^ cells, MHCII^-^ CD172A^+^ cells, MHCII^+^ CD172A^-^ cells, CD16^-^ CD172A^+^ cells and CD16^+^ CD172A^+^ cells. Variation in acute phase proteins was moderately (CRP) to highly (HAPT) heritable. Among the four traits, which measured the total number (MON) or proportions (MHCII^+^ CD172A^+^, CD16^+^ CD172A^+^ and CD16^+^ MHCII^+^) of monocytes, moderate to high 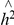, but not significantly different from zero, were estimated, except for CD16^+^ MHCII^+^ cells. Other haematological traits (RBC, HT, RDW and PLT) gave high h^2^ estimates, of which HT and PLT were significant.

### Genetic correlations among ITs

**Figure 3:**
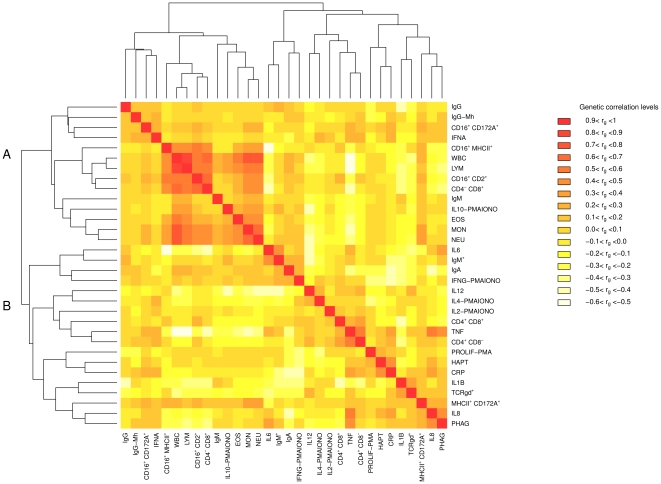
Heatmap of the genetic correlations between 32 ITs. The correspondence between colour scale and genetic correlation levels are presented on the right-hand side of the heatmap.

Pairwise genetic correlations are presented in Table S2 and illustrated in Figure 3. Genetic correlation estimates among most ITs were generally weak but a few high genetic correlations were observed. The number of positive genetic correlations (310) was higher than that of negative correlations (183), as already observed for phenotypic correlations. Positive genetic correlations were higher (in absolute values) than negative ones (Table S2, Figures 3 and S1). Only 28 (2.7% of the total number of correlations) and five 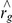 (0.4% of the total number of correlation estimates) were higher than 0.4 or lower than -0.4, respectively.

The unsupervised hierarchical clustering distinguished two main clusters of traits (Figure 3). The first cluster of 14 traits (Cluster A) could be divided into two groups: i) a group of four traits including one innate immunity cytokine (IFNA), two antibody levels (total IgG and specific IgG-Mh), and one FACS-characterized leukocyte subpopulation (CD16^+^ CD172A^-^) and ii) a group of 10 traits with nine hemogram-based cell counts or FACS-characterized leukocyte subpopulations (WBC, LYM, MON, NEU, EOS, CD16^+^ MHCII^+^, CD16^+^ CD2^+^, CD4^-^ CD8^+^ cells) and two cell activity traits (IgM and IL10-PMAIONO). The second cluster of 18 traits (Cluster B) is also subdivided into two groups of traits: i) a group of three different cell response traits (IL6, IgA and IFNG-PMAIONO) and one FACS-characterized leukocyte subpopulation (IgM^+^ cells), and ii) a group of nine cell response traits (IL12, IL4-PMAIONO, IL2-PMAIONO, TNF, PROLIF-PMA, HAPT, CRP, IL1B, IL8 and PHAG) and four FACS-characterized leukocyte subpopulations (CD4^+^ CD8^-^, CD4^+^ CD8^-^, TCRγδ^+^ and MHCII^+^ CD172A^+^ cells). In cluster A, the first group of 10 traits showed moderately to highly positive genetic correlations with each other. Indeed, 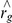 values greater than 0.4 were estimated between WBC, LYM, MON, NEU, EOS, CD4^-^ CD8^+^ and CD16^+^ CD2^+^ (NK) cells (Figure 3, Table S2). In cluster B, 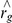
_values_ greater than 0.4 were estimated between i) TNF, IL8 and PHAG, and ii) CRP and HAPT (Figure 3, Table S2). Strong negative 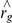 values (<−0.4) were found between a few traits from both clusters: i) TNF and three cell number traits (WBC, LYM, MON), ii) IL6 and CD16^+^ MHCII^+^, and iii) CRP and IgA.

## Discussion

### The measured ITs globally cover innate and adaptive immunity

The large-scale study reported here allowed us to estimate the genetic and phenotypic parameters of numerous ITs measured on pigs bred in the same environment. Innate immunity is represented by 25 traits, humoral-mediated immunity by five traits and cell-mediated adaptive immunity by 18 traits. We have also considered the total number of white blood cells and lymphocytes, and four other haematological traits (RBC, HT, RDW and PLT). Within cell-mediated immunity, we explored both Th1 and Th2 responses by measuring cytokine production. Figure 1 summarizes the traits that we selected to cover immunity globally. These traits include *in vivo* measures on blood such as quantification of cell populations by hemogram, dosage of circulating immunoglobulins and acute phase proteins, as well as *ex vivo* measures obtained after *in vitro* tests such as lymphocyte proliferation, phagocytosis capacity and cytokine production after blood stimulation. All these ITs have been widely studied in humans [25], [26]. The trait typology we have used (Table 1) follows a model based on a clear distinction between innate and adaptive immunity, which may be over-simplistic since both immune systems are closely interconnected [27], [28]. Monocytes are involved in innate immunity but are also antigen-presenting cells required for adaptive immunity, and cytokines such as IL12 are at the interface between innate and adaptive immunity. Similarly, the cell-mediated and humoral adaptive immunity subdivision is artificial. For example, IL4 is a cytokine produced by Th2 lymphocytes that is usually classified as part of adaptive cell-mediated immunity, whereas it is also involved in antibody production and thus adaptive humoral immunity. In addition, the conventional paradigm that CD4^+^ αβ T lymphocytes differentiate into Th1 and Th2 lineages expressing specific cytokines is collapsing. Indeed, recent studies have revealed that cytokine production by the different CD4^+^ T cell subsets (Th1, Th2, Th, Th17 and iTreg) is highly flexible, providing new insight into the Th cell plasticity [29]. Nevertheless, although schematic, the approach used in our report provides a comprehensive overview of genetic variation and co-variation across the entire immune spectrum in pigs (Figure 1).

### Phenotypic variation of ITs

Table 1 illustrates various ranges of phenotypic variation in the measured immune traits, with most having a CV over 0.5. Such variability has already been reported in large panels of healthy humans [26], and in previous studies on pigs [2], [16], [17], [30]. For instance, we substantiated the high level of variation of cytokine production previously reported for IL2 production and virus-induced IFNα production in a Swedish Yorkshire population [20]. For innate immunity-related cytokines and IL12, stimulation was performed with a mixture of LPS, PMA and ionomycin. IL8 was the cytokine produced with the highest levels followed by IL1B, TNF, IL12 and lastly IL6. These four cytokines are not expected to be expressed at similar levels at all time points after stimulation and a kinetic study would help to improve comparison of cytokine production levels. Weaker levels of adaptive IR cytokines are observed after LPS stimulation compared to CONA or PMAIONO. These differences could be related to the distinct modes of action of these molecules. Indeed, PMA, a plant-derived functional analog of diacylglycerol, in conjunction with ionomycin, a calcium ionophore produced by *Streptomyces conglobatus,* and CONA, a lectin originally extracted from the jack-bean *Canavalia ensiformis,* are known to be potent mitogens of blood lymphocytes [31], [32]. LPS, a major structural component of the outer membrane of gram-negative bacteria, which binds the CD14/TLR4/MD2 receptor complex and promotes the secretion of pro-inflammatory cytokines, has been extensively used to study innate immune response [33]. We have already shown that transcriptome modifications in peripheral blood mononuclear cells (PBMCs) differ between PMAIONO and LPS stimulation and that PMAIONO and LPS target different cells and cellular pathways [34]. The combination of PMA and ionomycin induces a stronger stimulation that may be related to a higher production of cytokines as detected in the present study. In addition, the lymphocyte proliferation induced by LPS is weaker than that observed with other stimulants, as expected.

### Variation in numerous ITs is genetically controlled

Our study provides the first heritability estimates for innate and adaptive cytokine production and for lymphocyte proliferation after PMA-ionomycin and LPS stimulations in pig. Pro-inflammatory cytokines appear to show less heritable variation than adaptive system-related cytokines. Among the adaptive system-related cytokines, estimated heritability was weakest for cytokines produced after LPS stimulation, except for IL2 production. Heritability estimates for lymphocyte proliferation after CONA, PMA and LPS stimulations were moderate and that of lymphocyte proliferation after CONA stimulation was comparable to the value obtained by Edfors-Lilja and colleagues [15]. Moderate to high heritability estimates for cell count traits from hemogram or FACS also confirmed those previously obtained for WBC, total lymphocytes, neutrophils, eosinophils and some leukocyte subsets (for CD4^+^ and CD8^+^ T lymphocytes, γδ T lymphocytes, CD11R1^+^, CD11R1^+^ CD8α^-^, CD11R1^+^ CD8α^+^ and CD16^+^ MHCII^+^) [15], [16], [17], [19]. Likewise, our results confirmed the high heritability estimate for phagocytosis [15]. Conversly, a lower heritability estimate for CRP than previously reported was observed [15], [16]. Overall, the heritability estimates for these traits appear robust regardless of populations, environments and protocols.

Some discrepancies exist between our heritability estimates and previous results for humoral-mediated adaptive ITs and some innate ITs. Indeed, in our study, B lymphocyte levels (IgM^+^ cells) are not significantly heritable contrary to other results [16], [17] and specific IgGs (IgG-Mh) have lower heritability estimates (0.12, 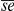  = 0.19) than previously reported (range from 0.27 to 0.45) for specific antibodies directed against other antigens [12]. Our estimated heritability for total IgG is higher (0.92, 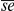  = 0.20) than in published reports [3], [15], [18], [19]. In addition, our estimated h^2^ for IFNα production is moderate to high (0.60, 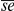  = 0.23), contrary to previous results (range from 0 to 0.08) [15], and for haptoglobin (0.55, 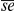  = 0.21) is higher than previously published (range from 0.14 to 0.23) [17], [19]. These discrepancies could be due to differences in the pig breeds and in environment factors but also to the absence of common standardised protocols between laboratories. In order to better qualify the phenotypes, protocol standardisation is needed.

Overall, we show that variation in both innate and adaptive ITs is under substantial genetic control (Figure 1; Tables 1 and 2). Similar heritability estimates for innate and adaptive ITs and also between cell number and cell response parameters were observed. Further, heritability estimates do not differ consistently between *in vivo* and *ex vivo* measures with no apparent bias due to phenotyping methods. These data also suggest candidate ITs for QTL mapping. Indeed, mapping studies have already started for total leukocyte count ([20], [21]; AnimalQTLdb, http://www.animalgenome.org/cgi-bin/QTLdb/index), mitogen-induced proliferation [20], antibody response [20], [22], cytokine production (IL2, IL10 and IFNγ) [23], [35], complement activity [22], and acute phase protein serum concentration [22].

### Phenotypic and genetic correlations illustrate the complementarity of the different ITs

Compared to the previously limited data on genetic correlations between ITs in pigs, our study provides a large-scale estimation of phenotypic and genetic correlations among 32 ITs. PCA results and correlation estimations highlight the weak phenotypic and genetic correlations between the different ITs, except mainly for cell subsets. No cluster of innate and adaptive ITs is revealed. These results illustrate that many of the ITs included in our study provide more or less independent potential clues for selecting for improved immunocompetence. Such complementarity is expected since innate immunity is in place or ready for activation prior to infection or antigenic stimulation and collaborates with adaptive immunity, which is induced by infection or antigenic stimulation. Nevertheless, a few highly positive genetic correlations have been detected between total number of white blood cells and some leukocytes subsets, and between some leukocyte subsets such as total number of lymphocytes, CD4^-^ CD8^+^ lymphocytes (which contains αβ CD4^-^ CD8^+^ lymphocytes and NK), and NK cells (CD16^+^ CD2^+^ cells). Phagocytosis, production of IL8 and TNF, two pro-inflammatory cytokines produced by monocytes and macrophages, were positively correlated with acute inflammatory phase proteins produced by hepatocytes i.e. CRP and HAPT. A high correlation was also found between CRP and HAPT. Clapperton and colleagues [17] have shown that phenotypic correlations between leukocyte subsets and acute phase proteins are weak (<0.2) and not significantly different from zero in agreement with our results. In contrast to our study, Clapperton and collaborators have not detected any significant genetic and phenotypic correlations between different leukocyte subsets except when one subset was nested in another [16].

### Toward the introduction of ITs in pig selection designs

Our results provide a framework for including ITs in multitrait selection for immunocompetence in pigs. Criteria for inclusion should take into account heritability, biological relevance, biological sensitivity and feasibility of measurement [25]. The weak genetic correlations between most ITs suggest that it will be difficult to choose only a few ITs to select for immunocompetence, and that a combination of many traits may be required [26]. The moderate to high heritabilities estimated for many traits together with the selection study on pigs carried out by Wilkie and colleagues a decade ago support the feasibility of selecting for immunocompetence [2], [13]. Chickens have also been successfully divergently selected for carbon clearance (phagocytic activity), high antibody response to Newcastle disease virus three weeks after vaccination (adaptive humoral IR) and wing web response to PHA (high cell-mediated immune response) for more than twelve generations [9], [36].

In order to include immunocompetence in selection for improved health, a major challenge will be to correlate variation in heritable ITs in healthy animals with inter-individual variability in response to various pathogens. Testing this hypothesis will be a key point for further use of ITs as indirect selection criteria in multitrait selection to improve resistance to disease. Some results on genetic and phenotypic relationships between immunocompetence and susceptibility to specific pathogens have already been reported in the literature for pigs. Among pigs selected for eight generations for high (HIR) or low (LIR) response based on an index of four cell and humoral-mediated immunity traits, an increased specific antibody response and lower polyserositis were observed in the HIR pigs compared to the LIR pigs after challenge with a novel pathogen, *Mycoplasma hyorhinis* [12], [37]. Thus, animals with a high IR level to unrelated challenges, as defined by Wilkie and colleagues [12], [37], have a better response to infection with *Mycoplasma hyorhinis*. However, HIR pigs develop more severe arthritis than LIR pigs. Indeed, the levels of humoral and cell-mediated adaptive ITs included in the Wilkie et al index induce the formation of immune complexes and/or the development of inflammatory responses, central to the pathogenesis of *Mycoplasma hyorhinis*-induced arthritis. Other correlation tests between ITs and response to various infections are needed. However, it is important to remain cautious with high responder animals, which could develop autoimmune pathologies or pathological iummne responses.

All the studies on immunocompetence and resistance to disease will have to be completed by estimation of genetic correlations with economically important traits already under selection. Negative genetic correlations have been reported between some ITs (monocytes, CD11R1^+^ cells) and average daily gain [16]. However a larger correlation study considering a higher number of ITs and pig performances is needed and is ongoing in our population. In the future, a more sustainable production system may require a compromise with a slight decrease in performance traded off for a gain in animal robustness. More studies are required to better understand the correlations between ITs and production traits and it is not established which levels of ITs would be good predictors for resistance to pathogens if any. In conclusion, our results show that variation in many ITs is under significant genetic control in pigs and these findings may provide insights in other species. Moreover, based on heritability and correlation estimations, some of the ITs that we have studied might be incorporated into selection schemes, provided they are associated with improved global health and do not exhibit strong antagonisms with other economically important traits.

## Materials and Methods

### Ethics Statement

Our experiment was conducted in accordance with the French national regulations for humane care and use of animals in research. No ethics approval was required for the vaccination and the collection of blood samples under the then current regulations. Experiments were performed under the individual license numbers 77-01 assigned to Marcel Bouffaud who was responsible for experiments in the test farm, and 78-16 assigned to a veterinarian, Dr Silvia Vincent-Naulleau. The experimentation agreement number for the test farm at le Rheu was A35-240-7.

### Animals and blood sampling

A total of 443 Large White pigs (castrated males, dam line) tested for performance traits in a pig test station (UE450, INRA, Le Rheu, France) was included in the study. The pigs were distributed in seven contemporary groups and belonged to 307 nuclear families obtained from 106 boars, with an average of 4.1 (+/− 1.7) piglets per boar. Animals were born and weaned in 16 different selection herds and arrived in the test station at five weeks of age with no prior vaccination. They were placed into pens of 30 piglets in a post weaning unit and vaccinated against *M. hyopneumoniae* (Stellamune, Pfizer, one injection) one day after their arrival in the test station, when 36.3 days old in average. All pigs were apparently healthy with no clinical sign of infection. All animals were sampled three weeks after vaccination. Blood samples were collected via the external jugular vein into tubes with or without anti-coagulants, according to further use.

### Serum and plasma collection

Blood collected in 10 mL tubes with no anti-coagulant was centrifuged at 3200 g for 15 min at 4°C. The serum was collected and stored at −20°C until use. Plasma was collected from blood sampled in heparinised tubes and stored at −20°C before use.

### Hemogram

Hemograms were measured with an MS4-5 counter (ElitechGroup, France) with blood sampled in EDTA tubes. Among a set of 18 traits, nine were included in the genetic analyses: total number of leukocytes, lymphocytes, monocytes, neutrophils, eosinophils, erythrocytes, platelets and hematocrit (Tables 1 and 2).

### Total and specific immunoglobulins

Total concentrations of immunoglobulin subsets were measured by ELISA as previously described [38]. Plasma samples were diluted 1∶6000, 1∶4000 and 1∶60,000 to detect IgM, IgA and IgG, respectively, in Tris–buffered saline and added to plates coated with immunoglobulin class specific pig antibody (Bethyl laboratories Inc., Interchim, France). The different subsets were detected with the appropriate peroxidase anti-pig IgM, IgA or IgG (Bethyl laboratories Inc.) and were quantified by reference to standard curves constructed with known amounts of pig immunoglobulin subsets. Anti-*M. hyopneumoniae* IgG titers were also measured by ELISA using a commercial kit (ELISA ID Screen® *M. hyopneumoniae* Indirect, IDVET, France). Absorbance was read at 450 nm using an ELISA plate reader (Spectra thermo, Tecan, NC, USA) and the Biolise 2.0 data management software.

### Haptoglobin and C reactive protein

Haptoglobin and C reactive protein levels were measured in pig serum by colorimetric tests (Phase Haptoglobin Assay, ABCYS Biologie, France) and ELISA assays (Porcine C reactive Protein Assay, ABCYS Biologie, France), respectively. Absorbance was read at 450 nm using an ELISA plate reader (MRX revelation, Dynex).

### PBMC purification

PBMCs were purified by density gradient centrifugation. A volume of 13 mL heparinised blood was added to Leucosept tubes (Greiner Bio-one, France) pre-filled with 17 mL Ficoll (Lymphocytes Separation Medium, Eurobio, France) and centrifuged at 1200 rpm for 35 min. PBMCs were collected at the ficoll interface and washed in 50 mL D-PBS without MgCl_2_ and CaCl_2_ (GIBCO, Invitrogen, France). Cells were then incubated in 2 mL BD Pharmlyse 1X (BD Biosciences, France) at room temperature.

### Leukocyte sup-populations by flow cytometry

Purified PBMCs were washed in 50 mL D-PBS without MgCl_2_ and CaCl_2_, incubated with 2 mL pig serum at 4°C for 20 min, washed again in 50 mL D-PBS without MgCl_2_ and CaCl_2_ and then washed in 50 mL S/W buffer (1 g/L NaN_3_, 10 g/L bovine serum albumin in PBS, pH 7.3) at a final concentration of 5.10^6^ cells/mL. 10^6^ cells were used for each antibody labelling. Single, double or triple staining was performed using monoclonal antibodies (mAbs) directed against i) CD2 (MSA4, isotype IgG2a, VMRD) and CD16 (MCA1971, isotype IgG1, Serotec), ii) CD4 (PT90A, isotype IgG2a, VMRD) and CD8α (PT81B, isotype IgG2b, VMRD) iii) TCRγδ (MAC320, isotype IgG2a, BD Biosciences Pharmingen), iv) IgM (PIG45A, isotype IgG2b, VMRD) and v) MHCII (MSA3, isotype IgG2a, VMRD), CD16 (MCA1971, isotype IgG1, Serotec) and CD172a (74-22-15A, isotype IgG2b, BD Biosciences Pharmingen). Briefly, PBMCs were stained with primary mAbs for 25 min at 4°C, washed in S/W buffer and stained with allophycocyanin-conjugated anti-mouse IgG1 (BD Biosciences, France), phycoerythrin-conjugated anti-mouse IgG2a (Southern Biotech, France), FITC-conjugated anti-mouse IgG2b (Southern Biotech, France), APC-conjugated anti-mouse IgG1 (BD Biosciences, France), phycoerythrin-conjugated anti-rat IgG2a (BD Pharmingen, France), or phycoerythrin-conjugated anti-mouse IgG2b (Southern Biotech, France). After washing in S/W buffer, cells were fixed in BD Cellfix solution (Becton Dickinson, Germany). Data acquisition and analysis were carried out with the FACScan and CELLQuest software (Becton Dickinson, UK).

### Assay of leukocyte-derived IFNα after *in vitro* viral stimulation of total blood

Synthesis of IFNα by leukocytes of pigs was tested *in vitro* by incubating diluted total blood in the presence of pseudorabies virus (PrV, *Suid Herpesvirus 1*)-infected, glutaraldehyde-fixed, PK15 cell monolayers, according to a protocol previously described for transmissible gastroenteritis virus [39]. Confluent PK15 cell monolayers grown in 24 well plates were infected by PrV at a multiplicity of infection of 20, fixed with 0.05% glutaraldehyde 8 h post-infection and washed with D-PBS and RPMI1640 before the addition of blood samples. For each animal, monolayers were incubated with diluted heparinized blood (270 µl of blood diluted 1∶5 in DMEM supplemented with antibiotics) for 18 h at 37°C. Plates were then centrifuged at 450 x g for 20 min at 4°C, and supernatants were collected and stored at -20°C. IFNα was assayed in the supernatants using a classical sandwich ELISA test as previously described [40].

### Production and dosage of IL1B, IL6, IL8, TNFα and IL12

Heparinized blood samples (400 µL) were fivefold diluted in 24-well plates in 1.6 mL RPMI 1640 medium (BioWhittaker, Belgium) supplemented with 10% heat-inactivated fetal bovine serum (QB perbio, UK), 2 mmol/L L-glutamine, 100 U/mL penicillin and 100 mg/mL streptomycin. For stimulation, a mixture of 10 ng/mL PMA (Sigma, France), 1 µg/mL ionomycin (Sigma, France) and 1 µg/mL LPS from *Escherichia coli* O111:B4 (Sigma, France) was added to the diluted blood. For mock stimulation, a volume of PBS equal to the volume of stimulation reagents was added to the diluted blood. After incubation at 37°C for 24 h, culture supernatants were collected by centrifugation at 450 g for 20 min and stored at −20°C before use.

The cytokines IL1B, IL6, IL8, TNF and IL12 were quantified using commercial ELISA tests (DuoSet ELISA development kits, R&D Systems, USA). For quantification of basal levels of cytokines in supernatants from mock-stimulated cells, the samples were not diluted for quantification. Supernatants collected from stimulated cells were diluted (1∶22 for IL1B and IL8, 1∶1 for IL6, 1∶10 for TNF and 1∶2 for IL12). All samples were tested in duplicates. Absorbance was read at 450 nm using an ELISA plate reader (MRX revelation, Dynex). Results were expressed as pg of cytokine/mL.

### Production and dosage of IL2, IL4, IL10 and IFNγ

Heparinized blood diluted 1∶5 in complete culture medium consisting of DMEM (Dulbecco's Modified Eagle Medium, Eurobio, France) supplemented with 5% fetal calf serum (Hyclone, Perbio, France), 2 mM L-glutamine, 100 U/mL penicillin and 50 µg/mL streptomycin (Eurobio, France) was stimulated with 10 µg/mL CONA (Sigma, France), or with 50 ng/mL of PMA (Sigma, France) and 1 µg/mL of ionomycin (Sigma, France) or 1 µg/mL LPS from *Escherichia coli* (Sigma, France). Cytokine content was measured in supernatants using ELISA tests as already described [41]. Briefly, purified fractions of anti-swine IL-2, IL-4, IFNγ (clones A150D 3F1, A155B 16F2 and A151D 5B8 respectively, Biosource, France) and IL10 (clone 148801, R and D System, France) were used as capture antibodies, in conjunction with the biotinylated anti-swine IL-2, IL-4, IL10 and IFNγ monoclonal antibodies (clones A150D 8H10, A155B 15C6 and A151D 13C5, respectively, Biosource, Clinisciences, France) or anti-swine polyclonal antibody (goat anti-porcine IL-10, R and D System, France). Streptavidin-horseradish peroxidase (Biosource) and TMB (Fermentas, MD, USA) were used for detection. Absorbance was read at 450 nm using an ELISA plate reader (Spectra thermo, Tecan, NC, USA) and the Biolise 2.0 data management software. Recombinant pig IL-2, IL-4, IL10 and IFNγ were used as standards. The detection limits were 700 pg/mL, 60 pg/mL, 90 pg/mL and 100 pg/mL for IL-2, IL-4, IL10 and IFNγ, respectively. Results were expressed as pg of cytokine/mL.

### Measurements of lymphocyte proliferation

Lymphocyte proliferation was performed in 96 well plates as already described [42]. Briefly, heparinized blood samples were diluted 1∶15 in complete culture medium consisting of DMEM (Dulbecco's Modified Eagle Medium, Eurobio, France) supplemented with 5% fetal calf serum (Hyclone, Perbio, France), 2 mM L-glutamine, 100 U/mL penicillin and 50 µg/mL streptomycin (Eurobio, France). For detection of unspecific lymphocyte proliferation, the diluted blood samples were seeded in 96 well plates (200 µL/well) and mock-stimulated for 48 h by incubation in culture medium (control wells), or stimulated for 48 h by incubation with the culture medium supplemented with either 10 µg/mL ConA (Sigma, France), or 50 ng/mL of PMA (Sigma, France) and 1 µg/mL of ionomycin (Sigma, France), or 1 µg/ml LPS (Sigma, France). Control wells remained unstimulated. After 48 h of incubation at 39°C, 0.5 µCi of ^3^H-methyl-thymidine (ICN, France) was added to each well. After another 24 h incubation period, the cells were harvested through glass-fiber filters (Whatman, United Kingdom) by means of an automatic harvester (Titerteck-Skatron, Molecular Devices, France). Incorporation of tritiated thymidine was measured with a Liquid Scintillation Beta Counter (Kontron Instruments, France). Results were expressed as a stimulation index of lymphocyte proliferation calculated as mean counts per min (cpm) of the triplicate cultures in stimulated culture/mean cpm in control non-stimulated culture.

### Data analysis

Preliminary statistical analyses were performed using R software [43]. In order to test if trait distributions deviated from Gaussian, a D'Agostino normality test was used (p = 0.05). Since most traits were not sampled from a Gaussian distribution, they were all normalized using a Box-Cox transformation except for phenotypes reaching the value zero, which were normalized using ln(1+x) transformation. Significant effects of age at the time of vaccination, of time of vaccination, of breeding unit and of time of experiment were detected for most traits by variance analysis taking into account these effects. Normed Principal Component Analysis (PCA, [44], dudi.pca function, ade4 package [45], R software [43]) on a subset of ITs adjusted for age at the time of vaccination, time of vaccination, breeding unit and experiment (Table 1) were performed using a linear model. Clusters of ITs were detected using the R package mclust for normal mixture modelling and model-based clustering [46]. It combines model-based agglomerative hierarchical classification, based on the classification likelihood, and the expectation-maximization (E–M) algorithm for maximum likelihood estimation of multivariate mixture models. Variance components, genetic parameters and their standard errors were estimated by the Restricted Maximum Likelihood (REML) method [48], using the WOMBAT software [49]. This is the reference method to estimate genetic parameters with a mixed model. Univariate and bivariate mixed linear animal models were employed to estimate heritability and genetic correlations, respectively. For the univariate analyses, the fixed part of the model included experiment time, age at the time of vaccination, vaccination time and herd of origin effects and the random part included a common litter environmental effect and direct genetic effects. In matrix notation
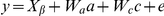



where y  =  the vector of observations; X_β_, W_a_ and W_c_ are known incidence matrix relating observations to fixed and random effects; ß  =  a vector of fixed effects and covariates; a  =  the vector of direct genetic effects; c  =  the vector of common litter environmental effects; and e  =  the vector of random residual effects. All random effects were assumed to follow a normal distribution with zero mean. For bivariate analyses, the same effects as for univariate analyses were taken into account in the fixed part of the model and a direct genetic effect was included in the random part of the model. 95% confidence intervals (95CI) were calculated for heritability (h^2^) estimates (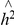). Heritability estimates have been classified: high (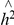
>0.4), moderate (0.1<
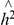
<0.4) or weak (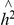
≤0.1). A graphical representation of the genetic correlations combined with a hierarchical clustering (euclidian distance, average link) was obtained with the heatmap function from the Bioconductor software [50]. Comparisons of heritability average between subsets of traits were tested using the Wilcoxon Mann*-*Whitney Test (significance threshold p-value = 0.05).

## Supporting Information

Figure S1 **Heatmap of the phenotypic correlations between 32 ITs**. The correspondence between colour scale and genetic correlation levels are presented on the right-hand side of the heatmap.
(TIF)

Table S1 **Additional details on the measurements of the ITs**.
(XLS)

Table S2 **Phenotypic and genetic correlations between 32 ITs**.
(XLS)
