# Supplementary figures and images for: Immunity Traits in Pigs: Substantial Genetic Variation and Limited Covariation
Source: PLoS One. 2011 Jul 29;6(7):e22717. doi: 10.1371/journal.pone.0022717 (PMC3146468; doi:10.1371/journal.pone.0022717)

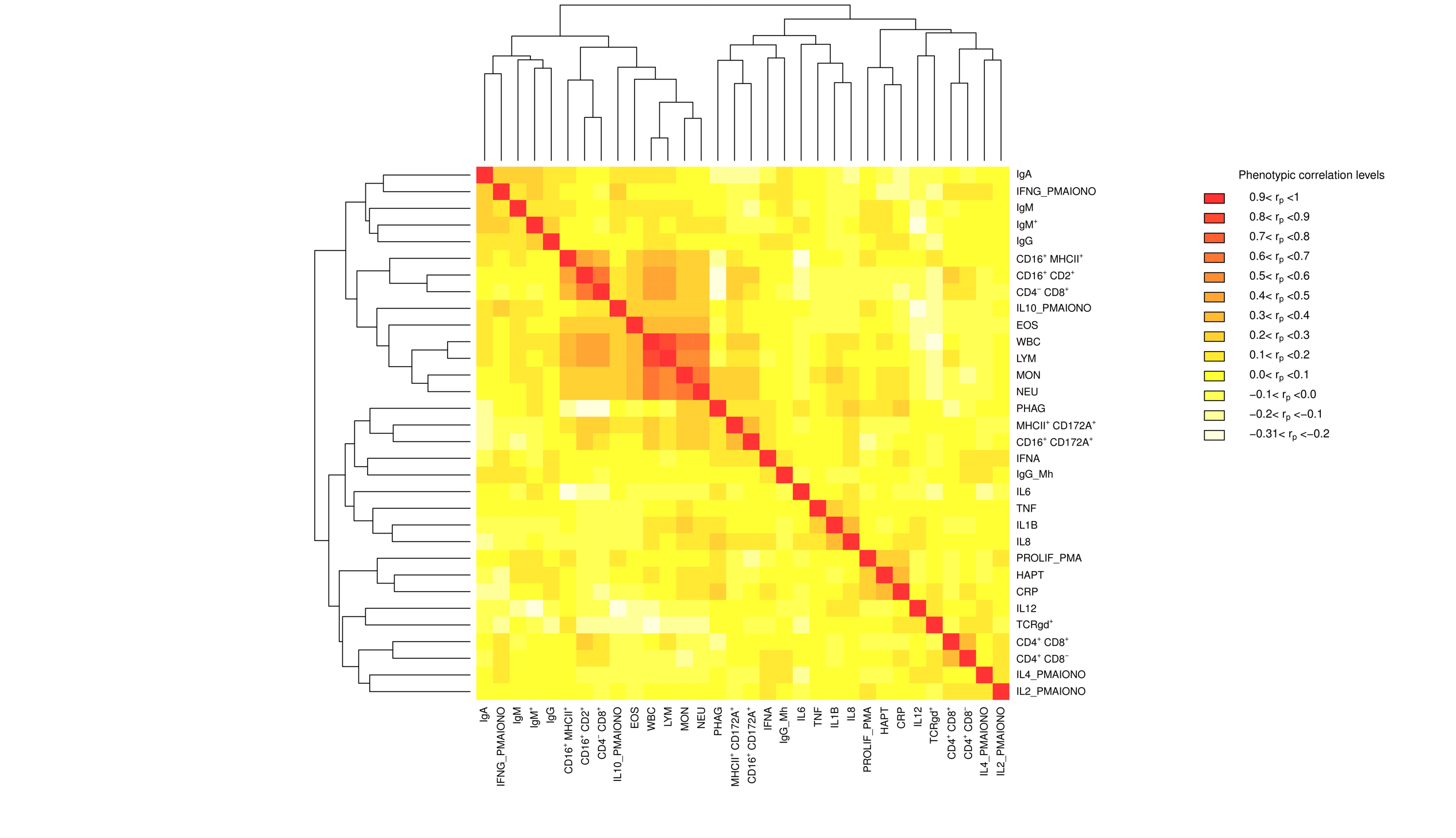

Supplement: Figure S1 — Heatmap of the phenotypic correlations between 32 ITs. The correspondence between colour scale and genetic correlation levels are presented on the right-hand side of the heatmap. (TIF) [file pone.0022717.s001.tif]
